# Symmetrical and Asymmetrical Interactions between Facial Expressions and Gender Information in Face Perception

**DOI:** 10.3389/fpsyg.2017.01383

**Published:** 2017-08-14

**Authors:** Chengwei Liu, Ying Liu, Zahida Iqbal, Wenhui Li, Bo Lv, Zhongqing Jiang

**Affiliations:** ^1^School of Education, Hunan University of Science and Technology Xiangtan, China; ^2^School of Psychology, Liaoning Normal University Dalian, China; ^3^College of Preschool and Primary Education, Shenyang Normal University Shenyang, China; ^4^Collaborative Innovation Center of Assessment toward Basic Education Quality, Beijing Normal University Beijing, China

**Keywords:** facial expression, facial gender, interaction, ERP, face perception

## Abstract

To investigate the interaction between facial expressions and facial gender information during face perception, the present study matched the intensities of the two types of information in face images and then adopted the orthogonal condition of the Garner Paradigm to present the images to participants who were required to judge the gender and expression of the faces; the gender and expression presentations were varied orthogonally. Gender and expression processing displayed a mutual interaction. On the one hand, the judgment of angry expressions occurred faster when presented with male facial images; on the other hand, the classification of the female gender occurred faster when presented with a happy facial expression than when presented with an angry facial expression. According to the evoked-related potential results, the expression classification was influenced by gender during the face structural processing stage (as indexed by N170), which indicates the promotion or interference of facial gender with the coding of facial expression features. However, gender processing was affected by facial expressions in more stages, including the early (P1) and late (LPC) stages of perceptual processing, reflecting that emotional expression influences gender processing mainly by directing attention.

## Introduction

Facial expressions and gender information are always intertwined in human faces. We perceive a difference between a crying male and a crying female because there is an interaction between facial expression information and gender information. Previous studies have provided evidence to support this idea; for example, participants were usually faster and more accurate in detecting angry expressions on male faces and happy expressions on female faces (Becker et al., 2007), and gender classification occurred faster with happy female faces than angry female faces (Aguado et al., 2009). Previous studies have also provided neurophysiological evidence of an interaction between facial expression and gender. An evoked-related potential (ERP) study revealed an interaction between facial expressions and gender in the face-sensitive N170 component (Valdés-Conroy et al., 2014). A functional magnetic resonance imaging (fMRI) study revealed that the left amygdala in female participants was more active in successfully remembering fearful female faces, while the right amygdala in male participants was more involved in the memory of fearful male faces (Armony and Sergerie, 2007).

The following two different hypotheses regarding the interaction between facial expressions and gender have been proposed: bottom-up processing and top-down processing. The bottom-up processing hypothesis posits that the interaction between facial expressions and gender is a result of an overlap between two types of information (Becker et al., 2007; Hess and Anemarie, 2010; Zebrowitz et al., 2010; Slepian et al., 2011). For example, both a male face and an angry face have a smaller brow-to-lid distance; meanwhile, happy expressions could have an increase brow-to-lid distance, which is more similar to the female facial features (Slepian et al., 2011). The top-down processing hypothesis posits that top-down information (e.g., gender stereotypes, such as women tending to smile more than men, and men expressing anger more frequently than women) is the cause of the interaction between facial expressions and gender (Fabes and Martin, 1991; Lafrance et al., 2003; Neel et al., 2012). Although these two hypotheses are contradictory, the effect on people’s responses are nearly identical. We named this effect the associated effect of facial expression and gender.

Although current theories of facial perception tend to agree that there is an interaction between facial expressions and gender processing, there are conflicting findings regarding the manifestation of this interaction. Gender information has been found to affect the categorization of emotional expressions, whereas emotional expressions did not affect the categorization of gender information (Atkinson et al., 2005; Karnadewi and Lipp, 2011). Gender classification was shown to be influenced by facial expression information, but expression classifications remain relatively unaffected by the facial gender (Wu et al., 2015). Some studies have shown no interaction between facial expressions and gender processing, supporting that independent routes exits for processing facial expressions and gender (Le and Bruce, 2002; Nijboer and Jellema, 2012).

Regarding the causes of the contradictory results regarding the interaction between facial expressions and gender, we speculated that in addition to the reasons noted by Karnadewi and Lipp (2011), e.g., expression type, experimental paradigm, stimuli, etc., the relative strength of the two types of information (e.g., expression vs. gender) could modulate their interaction. The intensity of the facial expression affected the accuracy of the expression recognition (Montagne et al., 2007; Hoffmann et al., 2010). Garner (1983) noted that, during a multiple dimensional stimuli processing, the dimension with slower speed of processing was more susceptible to the faster. Therefore, the asymmetric interaction between facial expressions and gender information might be due to a mismatch in their intensities. If the intensity of the two types of information was matched, their interaction would likely be symmetrical, which is one of the main hypotheses tested in the present study.

Although the mutual influence of gender and expression could be symmetrical if their intensities were matched, the precise stage of facial processing during which one type of information influences the other could be different because there are differences in the time course of gender and expression processing. Gender information was observed to be quickly and automatically processed using ERP technology, which was reflected by the N170 component, whereas during the later processing stages, gender information was no longer processed if it was irrelevant to the task (Mouchetant-Rostaing et al., 2000; Castelli et al., 2004; Tomelleri and Castelli, 2012). Emotion information processing is relatively faster than gender information processing in face perception processing (Wang et al., 2016); the effect of information processing appears as early as 100 ms from the onset of a stimulus, which is indexed on the P1 ERP component (Pourtois et al., 2004; Rellecke et al., 2012). Furthermore, the emotion effect was also observed in the late positive component (LPC) (Wild-Wall et al., 2008; Frühholz et al., 2009; Hietanen and Astikainen, 2013).

Using both an expression task and a gender task, the present study explores the mutual impact of expressions and gender when one type of information is task-relevant, while the other is task-irrelevant. Based on the above discussion, we hypothesize that during the gender classification task, the facial expression effect can occur as early as the P1 component, and the facial gender effect is hypothesized to occur during the N170 component in the expression classification task.

## Materials and Methods

### Participants

Upon obtaining the approval of the Ethics Committee at University, a recruitment advertisement was posted at the entrance to the University, which is visibly accessible to all students. Twenty right-handed undergraduate participants (11 males, 9 females; aged 18–22 years; *M* = 19.55, *SD* = 1.23) were recruited for the experiment. The participants reported no history of brain diseases, or chronically taking any medicine affecting brain activity.

### Material Evaluation and Selection

Twenty-three undergraduate participants (11 males, 12 females; aged 18–22 years; *M* = 19.84, *SD* = 1.25) were requested to rate gender and expression intensity information of 185 face images from CAPS (Chinese Affective Picture System) (Bai et al., 2005) on a 9-point scale. For the expression component, the participants were instructed to rate the faces according to how angry or happy the faces appeared (1 = *very angry*, 5 = *neither angry nor happy*, 9 = *very happy*). For the gender information, the participants rated how masculine or feminine the faces appeared (1 = *very masculine*, 5 = *neither masculine nor feminine*, 9 = *very feminine*). Although gender and expression information is different in nature, the evaluation of the intensity of the two types of information is comparable due to the use of the same participants and pictures.

According to on the above mentioned rating results, we selected 80 faces with a balanced gender and expression intensity. A paired samples *t*-test showed that there were no significant differences in the intensity between the two types of information (gender and expression) in the happy face pictures, *t*(39) = 0.73, *p >* 0.05, and the angry face pictures, *t*(39) = 0.38, *p >* 0.05. An independent samples *t*-test revealed no significant difference in the intensity of the gender information between the happy and angry faces, *t*(78) = 0.83, *p >* 0.05, or in intensity of the expression information between the female and male faces, *t*(78) = 0.20, *p >* 0.05. Descriptions of these evaluations are shown in **Table 1**.

**Table 1:** The intensity of the gender and expression information in each group of images.

	Happy face		Angry face	
		
Information type	Female face	Male face	Female face	Male face
Gender	8.14 ± 0.05	7.98 ± 0.05	7.88 ± 0.05	8.14 ± 0.05
Emotion	8.11 ± 0.05	8.06 ± 0.05	7.98 ± 0.05	8.01 ± 0.05


### Procedures

The participants were seated in a quiet room in front of a computer at a distance of approximately 90 cm from the monitor screen. The face stimuli were presented in the center of the screen. All participants completed two tasks (expression discrimination: happy vs. angry; gender discrimination: male vs. female). Half of the subjects were first asked to discriminate between the facial expressions (happy vs. angry). The participants responded by pressing the right and left mouse buttons. The participants were provided 5 min of rest after the expression task was completed, and then the participants were asked to discriminate between male and female faces. The other half of participants were tested in the reverse order. Each stimulus combination (for example, happy female) was presented three times in each block, thus providing 240 trials per block for a total of 480 trials. A 2 × 2 × 2 within-subjects design was used, with gender (male vs. female), expression (angry vs. happy), and tasks (expression discrimination vs. gender discrimination) as the two levels.

**FIGURE 1:**
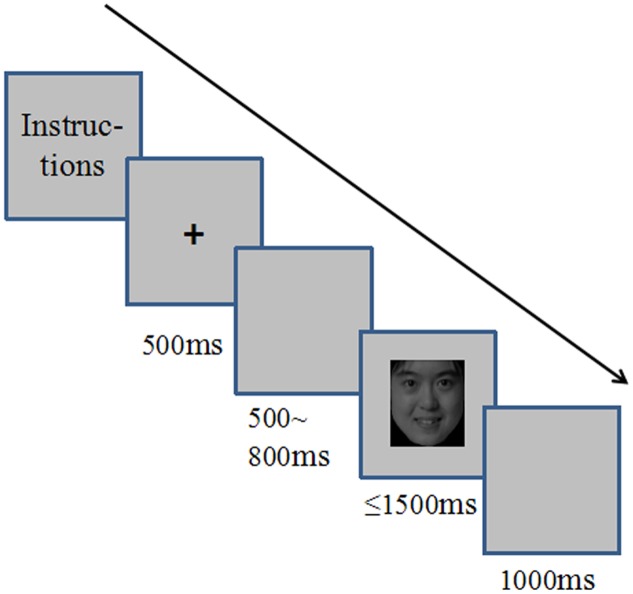
The sequence of events during an experiment trial.

The experiment included practice and formal sessions. During the practice session, the participants were presented with 16 pictures of faces and received feedback on their responses. Each trial began with a 500 ms fixation cross (“+”) at the center of the computer screen, followed by 500∼800 ms of a blank screen and the target face image. The face image remained on the screen until the participants responded or 1500 ms had passed (see **Figure 1**). The participants were instructed to judge the expression or gender of the face as quickly and accurately as possible. The participants responded by pressing keys. The assignment of the key mapping and task order was counterbalanced across the participants.

### Electroencephalogram (EEG) Signal Acquisition and Analysis

The EEG signals were sampled at 500 Hz from 64 cap-mounted Ag/AgCl electrodes referenced to the left mastoid and placed according to the expanded international 10–20 system (Neuroscan Inc., United States). The impedance was below 5 KΩ. The EEG was amplified using a bandpass filter of 0.05–40 Hz. Due to the interference of ocular potentials, horizontal eye movements were monitored by electrodes placed on the outside of each eye, and vertical movements were monitored separately by electrodes located above and below the left eye.

The EEG signals were re-referenced off-line to the common average of all scalp electrodes. Artifacts were rejected automatically if the signal amplitude exceeded ± 80 μV. Epochs of 1000 ms after the stimuli onset were computed with an additional 200 ms pre-stimulus baseline.

According to the ERP waveforms and previous studies (Itier and Taylor, 2002; Sato and Yoshikawa, 2007; Recio et al., 2011; Jiang et al., 2014), the amplitudes and latencies of each ERP component were derived from the averaged data obtained during the selected time windows over the electrode clusters as follows: P100 (100∼160 ms) and N170 component (160∼210 ms) over the electrode group including PO7, PO5, PO3, PO4, PO6, PO8, O1, OZ, and O2; LPC (350∼800 ms) over the electrode group including CP1, CPZ, CP2, P1, PZ, and P2.

## Results

### Behavioral Results

We tested the response accuracy using a 2 × 2 × 2 ANOVA, with task, expression and gender as the repeated-measures factors. The analysis did not find a significant main effect of task, *F*(1,19) = 3.79, *p >* 0.05, but a significant effect was found for facial expressions, *F*(1,19) = 19.07, *p <* 0.01, $\eta_{p}^{2}$ = 0.50, with a higher accuracy in the responses to the happy faces (*M* = 0.96, *MSE* = 0.01) than the responses to the angry faces (*M* = 0.93, *MSE* = 0.01). Importantly, a significant interaction was observed between facial expression and gender, *F*(1,19) = 5.49, *p <* 0.05, $\eta_{p}^{2}$ = 0.22. No task × facial expression × face gender interaction was found.

**FIGURE 2:**
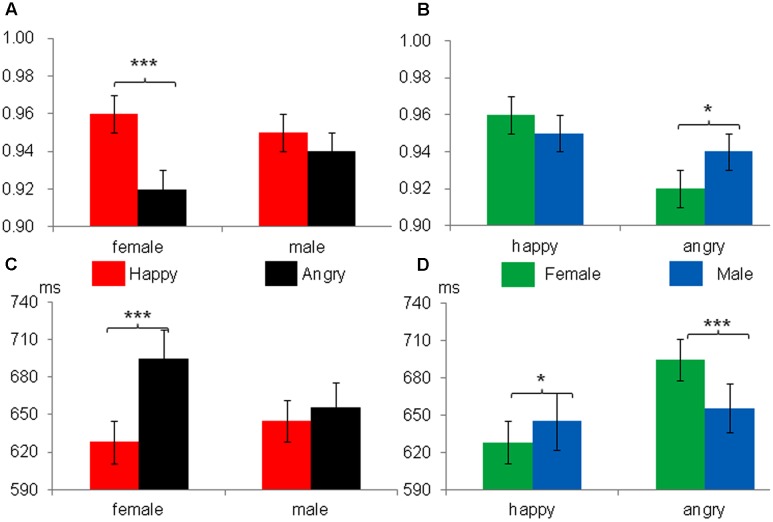
Participants’ accuracy **(A,B)** and response times **(C,D)** as a function of facial emotion and gender; the left images **(A,C)** reflect the effect of gender on expression processing; the right images **(B,D)** reflect the effect of expression on gender processing. ^∗^*p* < 0.05, ^∗∗∗^*p* < 0.001.

We further analyzed the interaction between facial expressions and gender from two perspectives. First, we explored the influence of expression on gender classification (see **Figure 2A**). The accuracy of judging the gender of a female face was significantly lower under the condition of angry faces (*M* = 0.92, *MSE* = 0.01) than under the condition of happy faces (*M* = 0.96, *MSE* = 0.01), *F*(1,19) = 40.49, *p <* 0.001, $\eta_{p}^{2}$ = 0.68. However, there was no significant difference in the recognition of male faces between the angry face (*M* = 0.94, *MSE* = 0.01) and happy face (*M* = 0.95, *MSE* = 0.01) conditions. Second, we explored the influence of gender on expression recognition (see **Figure 2B**), and the accuracy of judging an angry expression was significantly lower for female faces (*M* = 0.92, *MSE* = 0.01) than for male faces (*M* = 0.94, *MSE* = 0.01), *F*(1,19) = 4.64, *p <* 0.05, $\eta_{p}^{2}$ = 0.19. However, no significant differences were found in the accuracy of judging a happy expression between the female (*M* = 0.96, *MSE* = 0.01) and male (*M* = 0.95, *MSE* = 0.01) face conditions, *F*(1,19) = 1.12, *p >* 0.05, $\eta_{p}^{2}$ = 0.06.

A similar result was observed in the response time analysis. As shown in **Figure 1**, a significant main effect of facial expressions was found, *F*(1,19) = 32.24, *p <* 0.001, $\eta_{p}^{2}$ = 0.63, with faster RTs in response to happy expressions (636.51 ± 16.60 ms) than those in response to angry expressions (675.18 ± 21.29 ms). There was no significant effect of task, *F*(1,19) = 2.15, *p >* 0.05. There was a significant interaction between facial expressions and gender, *F*(1,19) = 36.13, *p <* 0.001, $\eta_{p}^{2}$ = 0.66. No task × facial expressions × gender interaction was found.

We further analyzed the interaction effect from two perspectives. First, regarding the influence of expression on gender recognition (see **Figure 2C**), the participants were slower to judge the gender of angry female faces (*M* = 694.56 ms, *MSE* = 23.16 ms) than they were to judge happy female faces (*M* = 628.03 ms, *MSE* = 17.01 ms), *F*(1,19) = 43.43, *p <* 0.001, $\eta_{p}^{2}$ = 0.70. However, there was no significant difference in judging the gender of male faces between angry (*M* = 655.81 ms, *MSE* = 19.91 ms) and happy expressions (*M* = 644.97 ms, *MSE* = 16.88 ms). Second, regarding the influence of gender on expression recognition (see **Figure 2D**), the participants were slower to classify the angry expressions on female faces (*M* = 694.56 ms, *MSE* = 23.16 ms) than those on male faces (*M* = 655.81 ms, *MSE* = 19.91 ms), *F*(1,19) = 29.17, *p <* 0.001, $\eta_{p}^{2}$ = 0.61. The results were opposite for the judgment of happy expressions as follows: the participants were slower to react to the male faces (*M* = 644.97 ms, *MSE* = 16.88 ms) than the female faces (*M* = 628.03 ms, *MSE* = 17.01 ms), *F*(1,19) = 6.2, *p <* 0.05, $\eta_{p}^{2}$ = 0.25.

### ERP Results

We performed a repeated-measures ANOVA using task (2: expression discrimination vs. gender discrimination), gender (2: male vs. female), and expression (2: angry vs. happy) as the within-subjects factors to analyze the amplitudes of P1, N170, and LPC separately. The results of the analysis revealed a significant task × facial expressions × gender interaction [P1 component, *F*(1,19) = 7.04, *p <* 0.05, $\eta_{p}^{2}$ = 0.27; N170 component, *F*(1,19) = 15.05, *p <* 0.05, $\eta_{p}^{2}$ = 0.44; LPC component, *F*(1,19) = 4.48, *p <* 0.05, $\eta_{p}^{2}$ = 0.19]. Therefore, we further explored the relationship between facial expressions and gender separately under the different task conditions.

### Gender Classification Task

A 2 (Gender) × 2 (Expression) repeated-measures ANOVA of the mean amplitude values of P1 and LPC revealed a significant facial expression × gender interaction, but no significant interactions were observed in the N170 component.

### P1 (100–160 ms)

**FIGURE 3:**
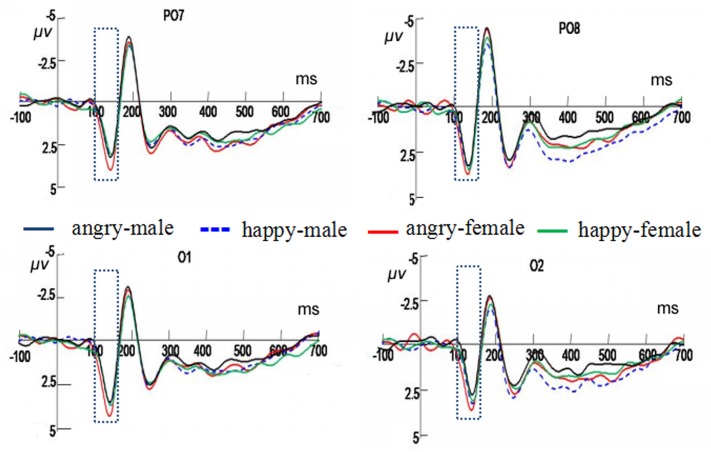
Averaged evoked-related potential (ERPs) at PO7, PO8, O1, and O2 in response to angry male faces, angry female faces, happy male faces, and happy female faces under the gender classification conditions. The time window of P1 is shown by the rectangle.

There was a significant interaction between facial expression and gender, *F*(1,19) = 5.48, *p <* 0.05, $\eta_{p}^{2}$ = 0.22. Further analysis revealed that higher amplitudes were elicited by the angry female faces (4.79 ± 0.59 μV) than by the happy female faces (4.14 ± 0.48 μV), *F*(1,19) = 4.49, *p <* 0.05, $\eta_{p}^{2}$ = 0.19, but no significant difference was observed in the gender classification of the male faces between the angry (3.96 ± 0.60 μV) and happy expression (4.17 ± 0.49 μV) (see **Figure 3**) conditions.

### LPC (350–800 ms)

**FIGURE 4:**
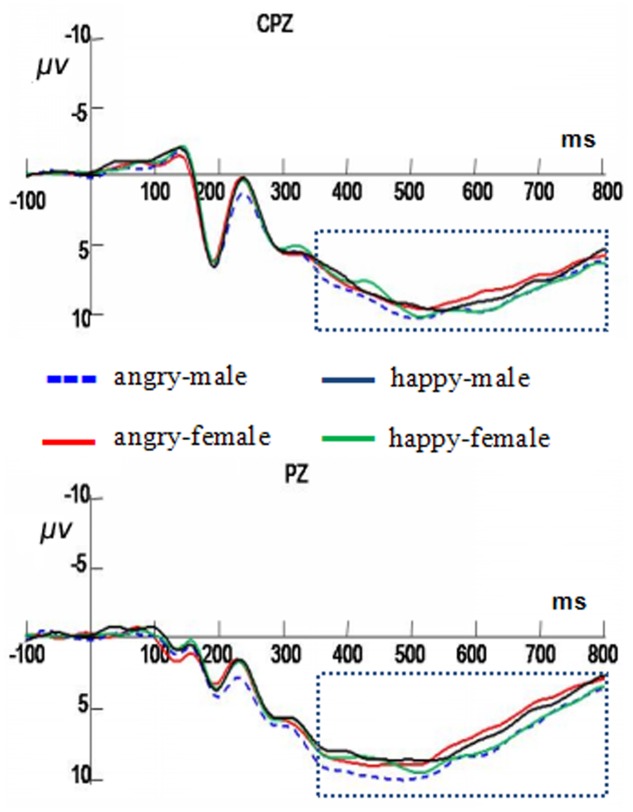
Averaged ERP sat Cpz and Pz in response to the angry male faces, angry female faces, happy male faces, and happy female faces under the gender classification conditions. The time window of late positive component (LPC) is shown by the rectangle.

The interaction between facial expression and gender was significant, *F*(1,19) = 5.66, *p <* 0.05, $\eta_{p}^{2}$ = 0.23. In the male faces, angry expressions elicited higher amplitudes (11.84 ± 1.03 μV) than the happy faces (11.08 ± 0.86 μV), *F*(1,19) = 11.03, *p <* 0.01, $\eta_{p}^{2}$ = 0.37. There was no significant difference in the female facial expressions (see **Figure 4**).

### Expression Classification Task

**FIGURE 5:**
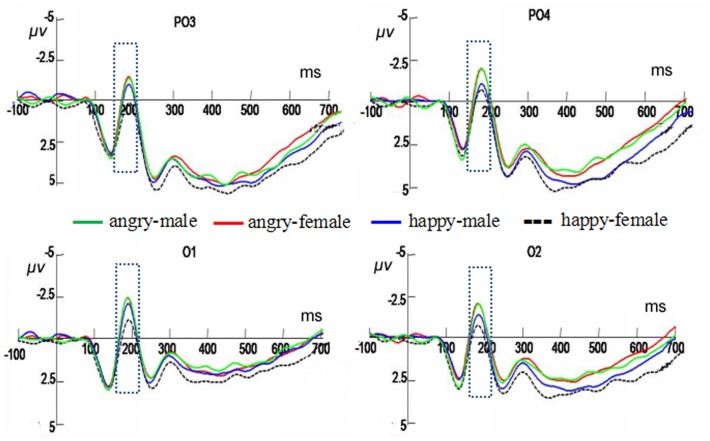
Averaged ERPs at PO3, PO4, O1, and O2 in response to the angry male faces, angry female faces, happy male faces, and happy female faces under the expression classification conditions. The time window of N170 is shown by the rectangle.

In the expression classification task, a significant interaction between facial expression and gender was obtained only in the N170 component, *F*(1,19) = 6.76, *p <* 0.05, $\eta_{p}^{2}$ = 0.26. Further analysis revealed that judging happy expressions in male faces elicited more negative amplitudes (-3.35 ± 0.97 μV) than that judging female faces (-2.68 ± 0.89 μV), *F*(1,19) = 4.59, *p <* 0.05, $\eta_{p}^{2}$ = 0.19. No difference was found in judging angry expressions between the male and female faces (see **Figure 5**).

## Discussion

In the present study, we selected face pictures with equivalent intensities of gender and expression information to perform an experiment that required participants to judge both expression and gender. The behavioral results revealed a significant interaction between gender and expression information in both tasks. Interestingly, the ERP results showed that the interaction between facial expressions and gender occurred during different stages of face processing because of the different tasks. The effect of facial expressions on gender processing was mainly reflected during the P1 and LPC components, while gender affected expression processing only during the N170 component.

### Symmetrical Interaction in Terms of the Existence of a Mutual Effect

The results of the behavior data in the present study revealed a symmetrical interaction between gender and facial expressions in face processing; thus, one type of information (i.e., gender or expression) processing was affected by the other (i.e., expression or gender). This result is inconsistent with previous studies (Atkinson et al., 2005; Karnadewi and Lipp, 2011) that reported that only gender information affects expression processing.

In a previous study (Atkinson et al., 2005; Karnadewi and Lipp, 2011), gender information unidirectionally affected expression information processing, which may have been due to the stronger intensity of the gender information relative to the emotional information because the gender classification was relatively faster than the expression classification. The present study pre-matched the intensity of the emotional information and gender information, which was also evidenced by the non-significant differences in the response time and accuracy of the participants’ performance during the expression judgment task and the gender judgment task. Therefore, when the two types of information are matched in intensity, a bidirectional influence of expression on gender and of gender on expression was found; therefore, we hypothesize that their interaction is symmetrical.

### Asymmetrical Interaction in Terms of Temporal Courses

The ERP data revealed that the interaction between facial expressions and gender differed along the time course of the face classification. The effect of gender was reflected in the N170 component, while the effect of facial expressions was mainly embodied in the P1 and LPC components.

After analyzing the details of these ERP results, we hypothesized that there were at least two underlying mechanisms. The first mechanism is the associated effect of gender and expression; that is, the congruence of the features of gender and expression (e.g., angry and male face vs. happy and female face) could facilitate their processing. Otherwise, if their features were incongruent (e.g., angry and female face vs. happy and male face), their processing could be hindered (Slepian et al., 2011). The second mechanism is the general effect of emotion, which usually appears as a negativity bias; thus, negative emotional stimuli could result in greater ERP components than positive stimuli (Huang and Luo, 2006). These two mechanisms could be added synergistically or cancel each other’s effect.

In the expression classification task, the happy male face elicited more negative N170 responses than the happy female face, but there was no significant difference in the N170 responses between the male face and female face when both faces were angry. In the present study, the happy expression was congruent with the female faces instead of the male faces. The inconsistent relationship between facial expressions and gender could increase the difficulty of face processing, hinder the participants’ performance, and increase the intensity of the responses in N170 (Rossion et al., 2000) in classifying happy expressions on male faces compared to classifying female faces. Regarding the classification of the angry expressions, although the participants’ response times and accuracy were different between the male and female faces, there was no consistency in the N170 component, which may be due to the joint effect of the two mechanisms mentioned above. Considering the associated effect of gender and expression, the features of expression and gender in the angry female faces were incongruent, but they were congruent in the angry male faces (Slepian et al., 2011), which increased the difficulty of the expression classification task for the angry female face. Thus, this incongruency could increase the N170 response to angry female faces relative to that to angry male faces; on the other hand, the emotion of anger may itself increase N170 as previous studies have reported a negativity bias (Batty and Taylor, 2003; Caharel et al., 2005; Huang and Luo, 2006). Therefore, there could be a ceiling effect on N170 that masks the differences between the male and female angry faces.

Regarding the gender classification task, the effect of expression first presented during the early ERP component of P1. The female face with an angry expression elicited more positive P1 than the happy expression, but no significant difference between the angry and happy faces was found for the male faces. These ERP results are consistent with the behavioral results, which revealed that the participants were slower and less accurate in classifying the gender when the expression was angry instead of happy only in female faces but not in male faces. P1 is considered to reflect the processing of low-level features in the extra-striatal visual cortex, and stimuli with special features usually induce more positive P1 amplitudes (Hillyard and Anllo-Vento, 1998). Considering that facial expressions produce distortions in the shape of individual facial features, such as lip raising or eye widening (Calder et al., 2001) and Slepian et al. (2011) noted that the female face naturally resembles a happy expression instead of an angry expression, we hypothesized that female faces would be more distorted by angry expressions than by happy expressions due to the incongruence, thus inducing larger P1 amplitudes in response to the angry female faces and increasing the difficulty of judging facial gender. Furthermore, the effect of emotion, which appeared as a negativity bias in this study, could contribute to the larger P1 amplitude in response to the angry female face than that to a happy female face.

Similarly, in the male faces, a happy expression could cause more distortion in the facial features than an angry expression because the features of male faces are more congruent with anger (Calder et al., 2001; Slepian et al., 2011). Therefore, the P1 amplitude in response to happy male faces should be larger than that in response to angry male faces; however, considering the negativity bias (Huang and Luo, 2006), an angry male face could elicit a larger P1 amplitude than a happy male face. Therefore, these two effects could play contradicting roles in modulating the amplitude of P1 such that the comparison between the P1 amplitude in response to the happy male faces and angry male faces became non-significant.

During the second stage of the expression effect on gender classification, which was reflected by the LPC, male faces with an angry expression elicited higher amplitudes than happy faces, but there was no significant difference in the LPC between the angry and happy expressions on female faces. The difference between the two expressions on male faces are similar to those observed in previous studies and display a negativity bias (Cacioppo and Berntson, 1994; Cacioppo et al., 1997; Wild-Wall et al., 2008; Frühholz et al., 2009; Hietanen and Astikainen, 2013). Meanwhile, the effect of expression on female faces was non-significant. We suspect this might be due to the congruency of expression and gender information in an angry male face, which could emphasize the angry information such that its effects could also be reflected during the LPC even under the condition of implicit processing (gender classification task). Angry female faces, however, demonstrate atypical facial expression features and thus could not be reflected during this stage. This result also confirms that the LPC, unlike the early ERP components (e.g., P1 and N170), most likely reflects the psychological meaning rather than the physical features of the stimuli.

### Limitations of the Present Study

Although the present study revealed differences in the interaction between gender and facial expressions using ERPs, there were certain confounding factors in the mechanism of the interaction. For example, the analysis could not directly distinguish the associated effect of gender and expression from the general effect of emotion, nor provide direct evidence differentiating the physical feature-based effects (i.e., through bottom-up processing) from the gender stereotypes-based effects (i.e., through top-down processing) in the interaction. The main cause of these limitations is that we did not separate the physical features from the concept of gender or expression in the stimuli. To resolve this confusion, a specific experimental paradigm (Fu et al., 2012) and stimuli (Ip et al., 2017) might be helpful.

## Conclusion

In summary, the present study revealed a symmetrical interaction in terms of the existence of a mutual effect between gender and expression processing during face perception when the intensity of both types of information was matched.

Furthermore, the present study also revealed asymmetry in the psychological and physiological mechanisms underlying the interaction between gender and expression information. The ERP results provided evidence that facial expression affected gender processing mainly by attracting the participants’ attention, which occurred during the early and late stages of face processing and was indexed by P1 and LPC; meanwhile, gender affected expression processing during the face structural encoding stage, as indexed by N170, by facilitating or interfering with facial expression structural information processing.

## Ethics Statement

This study was performed in accordance with the recommendations of the “Experimental guidelines, Liaoning Normal University Ethics Committee.” After being fully informed of the study, the participants provided written informed consent.

## Author Contributions

Conceived and designed the experiments: CL, YL, and ZJ. Performed the experiments: CL. Analyzed the data: CL and ZJ. Wrote the paper: CL, ZJ, ZI, WL, and BL.

## Conflict of Interest Statement

The authors declare that the research was conducted in the absence of any commercial or financial relationships that could be construed as a potential conflict of interest.
